# Dietary and Health Profiles of Spanish Women in Preconception, Pregnancy and Lactation

**DOI:** 10.3390/nu6104434

**Published:** 2014-10-20

**Authors:** Marta Cuervo, Carmen Sayon-Orea, Susana Santiago, Jose Alfredo Martínez

**Affiliations:** 1 Department of Nutrition, Food Sciences and Physiology, Center for Nutritional Research, University of Navarra, Pamplona 31008, Spain; E-Mails: mcuervo@unav.es (M.C.); ssantiago@unav.es (S.S.); 2 CIBERobn Physiopathology of Obesity and Nutrition, Institute of Health Carlos III (ISCIII), Madrid 28029, Spain; 3 Department of Preventive Medicine and Public Health, University of Navarra, Pamplona 31008, Spain; E-Mail: msayon@alumni.unav.es

**Keywords:** diet, health, lifestyles, score, preconception, pregnancy, lactation

## Abstract

The nutritional status and lifestyle of women in preconception, pregnancy and lactation determine maternal, fetal and child health. The aim of this cross-sectional study was to evaluate dietary patterns and lifestyles according the perinatal physiological status in a large sample of Spanish women. Community pharmacists that were previously trained to collect the data recruited 13,845 women. General information, anthropometric measurements, physical activity, unhealthy habits and dietary data were assessed using a validated questionnaire. Mean values and percentages were used as descriptive statistics. The *t*-test, ANOVA or chi-squared test were used to compare groups. A score that included dietary and behavioral characteristics was generated to compare lifestyles in the three physiological situations. The analysis revealed that diet quality should be improved in the three stages, but in a different manner. While women seeking a pregnancy only met dairy recommendations, those who were pregnant only fulfilled fresh fruits servings and lactating women only covered protein group requirements. In all cases, the consumption allowances of sausages, buns and pastries were exceeded. Food patterns and unhealthy behaviors of Spanish women in preconception, pregnancy and lactation should be improved, particularly in preconception. This information might be useful in order to implement educational programs for each population group.

## 1. Introduction

Nutrition in the periconceptional period, pregnancy and lactation is very important for the mother and child health status [1], and there is consistent evidence about the association between nutrition and lifestyles during pregnancy and health outcomes [2].

In preconception nutrition care, besides folic acid supplementation [3], it is important to have an adequate intake of iron, iodine, calcium, vitamins A and D, essential fatty-acids and dietary supplements, when necessary [4,5]. Besides, a greater adherence to the Mediterranean-type dietary pattern may enhance fertility [6]. On the other hand, high consumption of caffeine and alcohol, smoking, use of illegal drugs and mothers being overweight or underweight have been associated with higher difficulty conceiving [7,8].

Early nutrition factors might be involved in the long-term development of obesity [9], cardiovascular disease, diabetes and other non-communicable diseases [10], according to the developmental origin of health and disease (DoHad) theory [11]. Besides, maternal weight status during pregnancy has been linked to adverse birth outcomes, such as fetal growth, birth defects or preterm delivery [12,13]. Traditionally, nutritional epidemiology in pregnancy has centered on food consumption patterns [14,15,16,17] and specific macronutrients [18] or micronutrients inadequacy [19]. In general, pregnant women in developed countries are at risk of suboptimal intakes of folate, iron and vitamin D [20].

The diet quality and lifestyles of lactating mothers have an important role, not only in recovering nutritional status after pregnancy [21], but also in other health outcomes for mother and child [22]. A healthy and varied diet during lactation ensures a balanced maternal nutrition and the optimal concentration of some nutrients of human milk [23]. Specifically, concentrations of many vitamins, iodine and fatty acids [24] in human milk depend on or are influenced by maternal diet [23].

In Spain, a previous longitudinal study conducted in 80 women who were planning immediate pregnancy in preconception, were pregnant or were at six months postpartum concluded that dietary patterns did not change significantly from preconception to postpartum [25]. However, nutritional epidemiology studies in Spanish women at these physiological situations are scarce [7,26,27,28,29] and mostly are centered in iron [30] and iodine intake [31]. Besides, other studies have reported smoking habits [32,33,34], socioeconomic factors or self-care [27,35,36,37], mainly in Spanish pregnant women.

The period before, during and after pregnancy provides a great opportunity to assess nutritional status and to offer women practical advice to improve diet quality, become more physically active and to help them manage body weight effectively [38]. All women should be offered support to breastfeed their babies to increase the duration and exclusivity of breastfeeding [39]. In this context, it is necessary to assess nutritional status in order to provide an adequate dietary advice in preconception, pregnancy and lactation. To our knowledge, there are no previous studies that have examined at the same time these periods in Spain. Therefore, the two aims of the present work were to assess dietary patterns and unhealthy behaviors in a large sample of Spanish women in preconception, pregnancy and lactation and to propose a short healthy lifestyle score for these three life periods.

## 2. Experimental Section

### 2.1. Subject Recruitment

This cross-sectional, population-based study included 13,845 Spanish women in preconceptional pregnancy or lactation periods, who participated in a national plan of nutritional education, between November 2009 and March 2010. This program was conducted in order to provide information to these population groups about the importance of how women’s health behaviors could influence the development and health of their offspring. The study took place in 2794 pharmacies all over Spain, from both urban and rural areas. The information was collected through a validated questionnaire for this population [28]. Volunteers were recruited by community pharmacists (one pharmacist per pharmacy) and had a face-to-face interview with them. Subsequently, each potential volunteer was specifically asked if she would be willing to take part anonymously in the study. After ensuring that participants understood the information, only those who voluntarily accepted were enrolled. Voluntary completion of the questionnaire was considered to imply verbal informed consent. This study was conducted with the approval of the Spanish Council of Pharmacist (ref 14100127) on 29 June 2009, and the Board of the Institute of Food Sciences and Nutrition of the University of Navarra (ref 90), according to the guidelines laid down in the Declaration of Helsinki for anonymous surveys [40]. Additionally, this nutritional program has the recognition of Health Interest Activity by the Spanish Ministry of Health and Social Policy [41].

Prior to data collection, community pharmacists were recruited through the Spanish Pharmacists Council. To assure harmonization among interviewers, all of them received a training session by videoconference and an extensive document with explanations and a decision tree to interpret each question and other information needed about the survey [42]. This information was also available for all pharmacists involved in the study on a website [41]. Furthermore, this approach has been successfully applied in a previous study concerning an elderly population [43,44].

### 2.2. The Survey

The first question of the survey was the self-reported physiological status of woman: preconception, pregnancy or lactation. Pregnancy intention was assessed by response to the following question: “Time looking for pregnancy: less than 6 months, between 6 and 11 months or more or equal to 12 months”. Pregnant woman were asked if pregnancy status was medically confirmed. Type of pregnancy (unique, twins, triplets or more) and week of gestation were also recorded. We considered the lactation period as 6 months postpartum, and women were asked about the type of feeding in that period: breastfeeding, formula feeding or mixed.

To assess the nutritional status and food habits of women, information about anthropometric measurements, self-perception of nutrition and health educational level, physical activity, unhealthy lifestyle habits and diet data were inquired. The anthropometric measurements were taken by community pharmacists at pharmacies (actual weight and height). Body mass index (BMI) was calculated by dividing weight in kilograms by the square of height in meters. Self-reported pre-gestational weight was also registered in pregnant and lactating women. Anthropometric and physical activity data collection has been validated [28]. Anthropometric measurements validation was assessed by testing the accuracy of measurements collected by community pharmacists and comparing them to measurements collected by trained research staff. Physical activity information included the number of hours spent in lying, sitting and moving activities, paying special attention so that the sum of the hours of a day were 24 in total. We validated this physical activity information using as another gold standard physical activity questionnaire, previously validated for Spanish adults [28]. Subsequently, the individual activity factor was calculated for each subject applying activity factors set by the Food Agriculture Organization, World Health Organization [45].

Information about age, physical activity, educational level (no studies, primary, high school, university graduate), self-perception of health (very good, good, regular, bad, very bad, I do not know), self-perception of actual nutrition (very balanced, balanced, medium balanced, non-balanced, I do not know) and unhealthy lifestyles was obtained from self-reported information. Unhealthy lifestyle comprised smoking status (never, former/passive, actual), alcohol consumption (yes, no) and use of illicit drugs (never, former, actual). Participants were also asked if they followed a special diet (low calorie, low fat, low carbohydrates, low sodium diets or any type of vegetarian diets). Qualitative information on self-reported nutrient supplementation was also assessed with the baseline questionnaire, specifically: enriched milk in calcium or vitamins, folic acid/vitamin B_12_ supplements or enriched foods, iodine supplements/iodine salt, iron supplements or enriched foods, multivitamins and others (supplements or foods enriched in fiber, prebiotics or probiotics).

Concerning the diet, semi-quantitative information was assessed by a validated food frequency questionnaire (FFQ) in which basic foods were classified into twelve food groups, where 4 responses were possible: daily, weekly, monthly or never. After that, the daily frequency of food consumption was calculated. To assess the validity of food patterns information, we used as gold standard, the FFQ of the SUN project (Seguimiento Universidad de Navarra project), which has been validated for Spanish adult population [28]. The correlation coefficients obtained ranged between *r* = 0.4 and *r* = 0.6. The “questionnaire application guide” included information on the typical serving size for each basic food [44]. In order to estimate if the three groups fulfilled the dietary recommendations for Spanish women [46], nuts, legumes, fish, eggs and meat were grouped as protein group, and bread and rice, pasta and potatoes were grouped as the cereal group.

### 2.3. Data Collection

Community pharmacists, previously trained, collected the information by a face-to-face interview with the volunteers and introduced the answers in a specific platform located on the website created for this study (under a password). Data were refined, processed and analyzed in an anonymous and confidential way. From 14,972 participants that were initially included in the study, 1127 were excluded because of implausible or missing values on important variables. Therefore, the final sample for the analyses was 13,845.

### 2.4. Statistical Analyses and Additional Calculations

Mean values and standard deviations (SD) for continuous variables and percentages for categorical variables were used as descriptive statistics. Analysis of variance (ANOVA) or the chi-squared test (χ^2^ tests) were used to compare the baseline characteristics of the participants according to the physiological status.

Mean and SD were calculated for each food group in the three categories of physiological status. ANOVA tests were used to compare the means of consumption in the three categories, while *t*-tests were conducted to compare the mean of consumption of every food group with the dietary guidelines of the Spanish Society of Community Nutrition (Sociedad Española de Nutricion Comunitaria (SENC)) in each of the three periods [46].

We also generated a healthy lifestyle score to obtain an estimation of selected healthy behavior characteristics [47,48], based on the recommendations proposed by the SENC (Spanish Society of Community Nutrition) [46] and Mediterranean diet adherence [49]. The Mediterranean diet is a plant-based dietary pattern, where each food group is eaten in moderation, and the cultural lifestyle that accompanies this food pattern embodies a sense of community, physical activity and adequate rest. This diet includes vegetables, fruits, olive oil, legumes and nuts, in abundance; intake of fish, dairy products and wine with moderation; and small portions of meat and poultry and sweets [50]. Points were allocated as follows: participants earn a point if (1) olive oil was used as the principal fat; (2) the protein group was consumed ≥1/day; (3) cereals were consumed 3–6/day; (4) salads and vegetables were consumed at least 2 servings/day; (5) fresh fruits were consumed ≥2 servings/day; (6) sausages were consumed <1/day; (7) buns and pastries were consumed <2/week; (8) they were never smokers (actual smokers deduct one point); (9) they were never drug users (actual drug users deduct one point); (10) activity factor above the median; and (11) they were normal weight (BMI between 18.5 and 24.9 kg/m^2^) (weight was based on pre-pregnancy data). Therefore, the range of the scores that could be earned was from −2 to 11 points. ANOVA was used to compare the means of the healthy lifestyle score in the three categories.

Simple linear regression and then stepwise multiple linear regression analysis were used to evaluate the predictive strength of the lifestyle variables, using as the dependent variable the total healthy lifestyle score. All *p*-values presented are two-tailed; *p* < 0.05 was considered statistically significant. Analyses were performed using STATA/SE version 12.0 (StataCorp, College Station, TX, USA).

## 3. Results

The main characteristics of participants according to their physiological status are presented in Table 1. The mean age of the women included in the study was 31.8 (SD: 4.7) years. Women in the lactation period reported a higher activity factor, and a higher percentage of this group of women perceived their health status as very good. Furthermore, a higher percentage of this group were never smokers and never drug users. Only 48.9% of childbearing women were consuming folic acid (supplements or enriched food) and 14.1% multivitamins. As expected, women with the status of pregnancy consumed more supplements, such as iodine, folic acid and vitamin B_12_, iron, multivitamins and minerals or others.

Table 2 shows the means of the consumption of every food group, and we compared these means between the three periods and found differences between them almost in all of the food groups (*p* < 0.05), except in eggs, cereal group, bread and salad and vegetables Additionally, we compared the means of consumption of each of the groups with the recommendations from the SENC for each specific physiological status.

**Table 1:** Baseline characteristics according to physiological status (*n* = 13,845).

Characteristics	All	Pre-Conception	Pregnancy	Lactation	*p* ^a^
*N*	13,845	4471	5087	4287	
Age (years)	31.8 (4.7)	31.4 (4.8)	31.9 (4.6)	32.2 (4.6)	<0.001
Height (cm)	164.0 (6.1)	164.0 (6.1)	164.1 (6.2)	164.1 (6.1)	0.621
Weight (kg)	61.3 (8.9)	61.4 (9.6)	61.2 (8.4)	61.3 (8.6)	0.432
BMI (kg/m^2^)	22.8 (3.3)	22.9 (3.9)	22.7 (3.0)	22.8 (3.0)	0.926
Activity factor	1.43 (0.05)	1.43 (0.05)	1.41 (0.05)	1.45 (0.06)	<0.001
*Education level (%)*					
No studies	1.8	1.7	2.10	1.6	<0.001
Primary	16.0	13.2	16.8	18.1	
High school	35.1	34.1	34.9	36.4	
University graduate	47.1	51.1	46.2	43.9	
*Self-perception of health (%)*					
Very good	21.4	19.4	22.3	22.4	<0.001
Good	65.5	66.3	63.7	66.7	
Regular	11.3	12.1	12.1	9.6	
Bad	0.8	0.7	1.0	0.7	
Very bad	0.3	0.3	0.3	0.2	
Don’t know	0.7	1.1	0.7	0.7	
Self-perception of actual nutrition * (%)					
Very Balanced	45.9	44.8	47.3	45.5	<0.001
Balanced	38.7	36.6	39.6	39.7	
Medium balanced	13.0	15.9	11.0	12.4	
Non balanced	0.6	0.7	0.4	0.9	
Don’t know	1.8	2.1	1.7	1.5	
*Tobacco*					
Never	63.7	56.3	67.1	67.4	<0.001
Former/passive	22.9	23.2	23.9	21.3	
Actual	13.4	20.4	9.0	11.4	
*Alcohol*					
No	67.3	51.0	75.4	74.6	<0.001
Yes	32.7	49	24.6	25.4	
*Illicit Drugs*					
Never	97.8	96.9	98.2	98.4	<0.001
Former	1.3	1.6	1.3	1.1	
Actual	0.9	1.5	0.5	0.5	
Special Diets **					
No	85.0	83.8	85.7	85.4	0.021
Yes	15.0	16.2	14.3	14.6	
*Diet supplementation (yes %)*					
Enriched milk with calcium/vitamins	24.1	21.1	24.9	26.2	<0.001
Folic acid/vitamin B_12_	49.5	48.9	74.7	20.4	
Iodine/Iodine salt	30.5	26.1	41.3	22.3	
Iron	32.5	16.0	46.1	33.7	
Multivitamin and minerals	21.3	14.1	26.7	22.4	
Other supplements	39.7	35.9	48.9	32.7	
No supplementation	18.3	26.1	5.8	25.2	
*Main fat consumed*					
Olive oil	92.2	91.2	92.9	92.4	0.008
Others	7.8	8.8	7.1	7.6	

* Women were asked how they considered their actual nutrition in comparison with other women in their same physiological status. ** Including low-calorie, low-sugar, low-fat, low-salt, vegan, lacto-ovo vegetarian and others. Values are expressed as the mean (SD), unless otherwise stated. ^a^ Continuous variables were compared using analyses of variance. Categorical variables were compared using the chi-squared test.

**Table 2:** Mean (SD) of daily serving consumption in women according to physiological status.

Servings/Day	Physiological Status						*p*
	Preconception		Pregnancy		Lactation		
	Mean (SD)	SENC ^†^	Mean (SD)	SENC ^†^	Mean (SD)	SENC ^†^	
*N*	4471		5087	2	4287	2	
Protein group	1.96 ^‡^ (1.64)	2	1.96 ^‡^ (1.18)		1.98 (1.13)		<0.001
Meat	0.58 (0.58)		0.59 (0.51)		0.61 (0.50)		<0.001
Fish	0.42 (0.50)		0.44 (0.47)		0.43 (0.40)		<0.001
Eggs	0.36 (0.31)		0.34 (0.23)		0.35 (0.27)		0.10
Legumes	0.32 (0.50)		0.33 (0.39)		0.32 (0.39)		0.001
Nuts	0.27 (0.56)		0.27 (0.48)		0.26 (0.52)		0.04
Cereal group	2.39 ^‡^ (1.56)	3	2.38 ^‡^ (1.51)	4	2.42 ^‡^ (1.53)	4	0.34
Bread	1.72 (1.23)		1.71 (1.23)		1.73 (1.22)		0.78
Rice, pasta and potatoes	0.66 (0.71)		0.67 (0.68)		0.70 (0.73)		0.01
Dairy	2.00 (1.19)	2	2.26 ^‡^ (1.26)	3	2.30 ^‡^ (1.33)	4	<0.001
Salad and vegetables	1.22 ^‡^ (1.00)	2	1.26 ^‡^ (1.01)	2	1.24 ^‡^ (0.98)	2	0.10
Fresh fruits	1.82 ^‡^ (1.33)	2	2.06 (1.36)	2	1.93 ^‡^ (1.34)	2	<0.001
Sausages	0.46 * (0.58)	Occasionally	0.36 * (0.50)	Occasionally	0.43 * (0.54)	Occasionally	<0.001
Buns and pastries	0.43 * (0.88)	Occasionally	0.35 * (0.59)	Occasionally	0.40 * (0.67)	Occasionally	<0.001

^†^ Recommendations from the SENC (Sociedad Española de Nutricion Comunitaria (Spanish Society of Community Nutrition)), using as a reference the minimum serving of recommended range for each group. * Exceeded the recommendation from SENC. ^‡^ Did not reach the recommendation from SENC.

Women in a preconceptional status did not reach the recommendation for consumption in the following food groups: proteins, cereals, salad vegetables and fresh fruits. On the other hand, women who were pregnant did not reach the recommendation for proteins, cereals, dairy and salad and vegetables. Finally, lactating women did not reach the recommendation for cereal, dairy, salad vegetables and fresh fruits. All of the women exceeded the recommendation for sausage and bun and pastry consumption.

**Figure 1:**
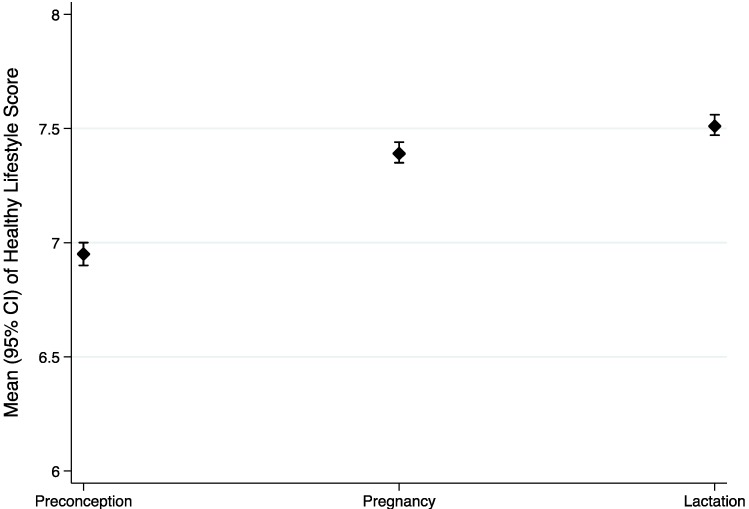
Mean and 95% confidence interval of healthy lifestyle score in each physiological status.

The definition of the healthy lifestyle score is presented in Table 3. The mean healthy lifestyle score was 7.29 (SD: 1.65) points. When we compared the mean of the score in each group of women, we observed a statistically significant difference between groups, finding a higher score in those women who were in the lactation period 7.51 (95% CI: 7.47–7.56). In Figure 1, the means and the 95% CI of the healthy lifestyle score in each group of women are shown.

In the stepwise multiple linear regression analysis, the factors that were more associated with the healthy lifestyle score were tobacco, fresh fruits, salad and vegetables, buns and pastries and activity factor. The coefficient of determination of these variables in the simple regression model was *R*^2^ = 0.299 (tobacco), *R*^2^ = 0.267 (fresh fruits), *R*^2^ = 0.209 (salad and vegetables), *R*^2^ = 0.156 (buns and pastries) and *R*^2^ = 0.112 (activity factor) (Table 4).

**Table 3:** Definition of the healthy lifestyle score.

Characteristics	Condition to Score	Points
Olive oil as principal fat	Yes	1
Protein group ^1,4^	≥1 serving/day	1
Cereal group ^2,5^	3–6 servings/day	1
Salad and vegetables ^6^	≥2 servings/day	1
Fresh fruits ^7^	≥2 servings/day	1
Sausages ^8^	≤1 serving/day	1
Buns and pastries ^3,9^	<2 servings/week	1
Tobacco	Never	1
	Former	0
	Actual	−1
Illicit drugs	Never	1
	Former	0
	Actual	−1
Activity factor	Above the median	1
Pre-gestational BMI	18.5–24.9 kg/m^2^	1
Total		−2 to 11

^1^ The protein group included: meat, eggs, fish, legumes and nuts. ^2^ The cereal group included: bread, rice, pasta and potatoes. ^3^ The buns and pastries group included all commercial pastries, sweets, doughnuts, *etc.* ^4^ A serving size for the protein group was defined as: one egg, 100–125 g of meat, 125–150 g of fish, 30 g of nuts, 60–80 g of legumes. ^5^ A serving size for the cereal group was defined as: 40–60 g of bread, 60–80 g of rice or pasta and 150–200 g of potatoes. ^6^ A serving size of salad or vegetables was defined as: 150–200 g of salad or 200 g of cooked vegetables. ^7^ A serving size of fruit was defined as: 120–200 g of fruit or 200 mL of natural juice. ^8^ A serving size of sausages was defined as 50 g. ^9^ A serving size of buns and pastries was defined as 50 g.

**Table 4:** Proportion of total variability in the healthy lifestyle score (*R*^2^) explained by individual items.

Predictors *	Simple Linear Regression			Stepwise Regression (Cumulative *R*^2^)
	Preconception	Pregnancy	Lactation	Total
*N*	4471	5087	4287	13,845
Healthy Score	*R*^2^	*R*^2^	*R*^2^	*R*^2^
Tobacco	0.340	0.254	0.268	0.299
Fresh fruits	0.256	0.274	0.265	0.512
Salads and vegetables	0.175	0.238	0.226	0.614
Buns and pastries	0.157	0.167	0.148	0.694
Activity factor	0.125	0.121	0.098	0.793
Pre-gestational BMI	0.102	0.060	0.076	0.855
Olive oil	0.077	0.071	0.070	0.879
Cereal group	0.054	0.076	0.076	0.947
Illicit drugs	0.068	0.054	0.044	0.964
Sausages	0.036	0.024	0.021	0.977
Protein group	0.026	0.026	0.022	1.00

* All of the predictors of the model were statistical significant in the stepwise regression.

## 4. Discussion

This cross-sectional study examined the healthy behaviors and dietary patterns of 13,845 Spanish women in preconception, pregnancy or lactation situations. Results revealed that a high proportion of women in these periods had a very good or good self-perception of their health and diet, but their dietary patterns and health behaviors are not optimal.

The recommendation of folic acid supplementation was not completely fulfilled in preconception: only 48.9% and 14.1% of childbearing women declared that they were consuming folic acid supplements and multivitamins, respectively. Previous investigation on the Infancia y Medio Ambiente (INMA) cohort showed that women initiate folic acid supplementation after the recommended period [51]. In Spain, according to a study published in 2007 by the Spanish Collaborative Study of Congenital Malformations [52], trends in folic acid supplementation before pregnancy have increased form from 9% to 17.4%, and the results of the Spanish Autonomic Regions were quite similar. Furthermore, the last national survey of the dietary intake of Spain ENIDE, 2010–2011 [53], reported that folic acid intake in women from 18–25 years was 234.2 µg/day and from 25–44 years was 265.04 µg and concluded that these results seem to be of concern, especially in women of childbearing age, due to the risk of neural tube defects. The preconceptional use of folate supplements varies with education and awareness of the importance of folic acid intake among women of reproductive age, the healthcare system, access to and/or distribution of pre-pregnancy folic acid supplements and/or fortification of staple foods with folic acid [54]. In 2004, a review found evidence for suboptimal use of periconceptional folic acid supplements globally [55]. Comparing to other European countries, recent studies reported different rates in France, where only 14.8% women had started folic supplementation before conception [56], 8%–25% in Poland [57] and 64% in Ireland [58]. In our sample, preconceptional women did not reach the recommended consumption of cereals, fruits and vegetables. Consumption of protein group was also slightly lower than dietary guidelines. Recent results of the Australian Longitudinal Study on Women’s Health [17] found no evidence that women trying to conceive consume greater intakes of nutrient-rich foods prior to conception, and those who met key nutrient recommendations generally consume more fruit and dairy. Thus, these authors suggest that dietary guidelines should be revised for reproductive-aged women. In Spain, nutritional epidemiology on dietary patterns in childbearing women is scarce, but Cuco *et al.* (2006) [25] observed that diet in preconception does not vary significantly in pregnancy and postpartum.

Therefore, food intake information should be recorded in women who are planning a pregnancy in order to give early and effective nutritional education for a healthy pregnancy. Furthermore, previous investigations concluded that a healthy diet (such us the Mediterranean-type diet) seems to be an efficient alternative means of enhancing fertility [7] and may protect against being overweight and obesity during pregnancy [59]. Besides, it has been reported that women with a high adherence to a Mediterranean diet in early pregnancy had a significantly lower risk of delivering a fetal growth-restricted infant with respect to weight [29].

Concerning lifestyles, a higher percentage of women in preconception declared unhealthy habits, such as smoking and consumption of alcohol and illicit drugs (even occasionally), compared to pregnancy and lactation. Although the smoking rate has decreased in relation to previous studies in Spain [7,32,34], it is still high, even when pregnancy is confirmed [27,32,33,60]. Smoking women are more likely to be infertile and have an increased risk of miscarriage [8]. Notwithstanding this evidence and the global recommendation of folic acid supplementation in preconception, as well as the well-known negative influence of smoking, excess weight and other health risk behaviors on fertility and birth outcomes, there is necessarily more individual education and intervention in preconception care [2,4,61], especially among women with lower education level [62].

Previous investigations in Spain revealed that pregnant women have evidenced inadequate consumption of cereals and vegetables [27,35,60], which coincides with our results. Nevertheless, when comparing birth cohort studies in all of Europe, pregnant women in Spain consumed more vegetables, fruits and seafood [16]. Besides, the essential nutrient content of fruit, vegetables and grains (whole grains) increases fiber intake, which may help to alleviate constipation, a common complaint during pregnancy [2]. Furthermore, in our sample, the consumption of foods of the protein group and dairy were lower than Spanish dietary guidelines [46]. On the other hand, the percentage of pregnant women consuming iodine supplements was lower than previously reported [27]. Routine supplementation of iodine during pregnancy is widely recommended [63]. Studies conducted in Spain confirm that most women are iodine deficient during pregnancy and lactation and recommend iodine supplements in these stages, even also in preconception [64].

In our sample, lactating women did not meet the recommendations of cereals, vegetables, fruits and dairy. To our knowledge, there are no available specific studies on food patterns in lactating women in Spain, although Sanchez *et al.* [65] found that only 36% of lactating women meet energy, calcium and vitamin D recommendations. In this sense, it has been reported that energy intake increased immediately after birth, but six months after delivery, energy and nutritional intake decrease compared to preconception [66]. Thus, postpartum women should be advised to replenish nutritional stores, return to a healthy weight and prevent problems in subsequent pregnancies, as well as be encouraged to increase the consumption of whole grains, fruits and vegetables [2].

Additionally, in all cases, the recommended servings for sausages, buns and pastries were exceeded. These foods are usually high in saturated fatty acids or sugars, which should be limited in the diet, even more in pregnancy and lactating women, according to Spanish dietary guidelines [46].

Moreover, we devised an 11-item score to summarize several factors that are well known to be associated with healthier lifestyles, following the Mediterranean diet model [49]. Our results showed that women with the status of lactation and pregnancy had healthier lifestyles than women in with status of preconception. This outcome could be explained, because pregnancy is a period when the majority of women are in close contact with physicians, and they are more receptive to health messages [67]. However, we considered that better health advice should be given to all women in order to improve their dietary habits and lifestyles. Furthermore, it has been found that early antenatal health promotion workshops on lifestyle behaviors had a great impact on maternal and infant health outcomes [47]. We have to take into consideration that women with the status of preconception that were included in our study were all seeking to become pregnant. Recently, a new evidence-informed framework for maternal and new-born care [68] has been proposed, which highlights the components of a health system needed by childbearing women, including information, education and health promotion, for example on maternal nutrition, family planning and breastfeeding promotion.

In this context, a recent review by Anderson *et al.* [6] concluded that time to pregnancy might be impacted by factors that are modifiable, some of which have an effect that is conclusive on reproductive health, such as body weight, intake of folate and smoking. With regard to other factors, such as alcohol and caffeine, the literature is inconsistent. Therefore, health advice should be given to this population focusing on those modifiable factors.

Our study has certain limitations. Firstly, the cross-sectional design of our study did not allow us to measure behavior changes in participants over time. Besides, the origin of the recruitment (pharmacies) and the voluntary participation of the women may have resulted in selection bias, the sample not being totally representative, as women using pharmacies are expected to be aware of the importance of self-care and may have a higher education level and economic status. In Spain, all population groups have access to the national health system and to free (partial or total) drugs issued in the pharmacy. In particular, some prenatal supplements are partially financed, but multivitamins are not. Thus, although the results are not totally generalizable, they give valuable information about food patterns and lifestyles in a large sample of Spanish women (*n* = 13,845).

Another potential limitation is that the large number of examiners might cause an inter-observer variation; however, an effort was made to minimize this. Thus, all of the pharmacists were trained on the protocol interview, including an application guide to the questionnaire with detailed information about each item and a decision tree to interpret answers in each case [42]. Furthermore, a joint videoconference explaining the study was simultaneously broadcast to every provincial pharmacy college, and a website [41] was available for all pharmacists involved in the study to support consistency among interviewers. This kind of training session for health professionals usually has a positive impact on reflecting nutritional issues [69,70].

In addition, we were not able to present macronutrient and micronutrient intakes, because the FFQ includes only 12 items, so we assessed major food and supplementation habits in women, but not nutrient intake adequacy. Despite these limitations, this study adds interesting information about anthropometric measures, food patterns, physical activity (using a validated questionnaire) [28], as well as health behaviors in a large sample of Spanish women.

## 5. Conclusions

The present study suggests that food patterns and unhealthy behaviors in Spanish women in preconception, pregnancy and lactation should be improved, particularly in preconception.

Regarding dietary habits in the three subgroups, most of the food groups analyzed were not consumed within the recommendations of the Spanish dietary guidelines. On the other hand, women seeking pregnancy had, in general, worse health related habits. This information might be useful in order to implement educational programs for each population group. However, specific studies on micronutrient intake are needed to complement these data.
